# Cross-cultural adaptation and psychometric properties of the Chinese version of the Orthorexia Nervosa Inventory

**DOI:** 10.3389/fnut.2024.1491544

**Published:** 2025-01-23

**Authors:** Xinzhang Sun, Ying Lu, Chengping Jian, Hanqing Zhang

**Affiliations:** ^1^Health Science Center, Yangtze University, Jingzhou, Hubei, China; ^2^Department of Nursing, Union Hospital, Tongji Medical College, Huazhong University of Science and Technology, Wuhan, Hubei, China; ^3^Department of Nursing, Sixth Affiliated Hospital of Kunming Medical University, Yuxi, Yunnan, China; ^4^Faculty of Public Health, Mahidol University, Bangkok, Thailand

**Keywords:** orthorexia, psychometric properties, eating disorders, factor analysis, scale

## Abstract

**Background:**

Orthorexia nervosa refers to an unhealthy preoccupation with maintaining a perfect diet, which is marked by highly restrictive eating habits, rigid food rituals, and the avoidance of foods perceived as unhealthy or impure. In recent years, the Orthorexia Nervosa Inventory (ONI) has gained recognition as a promising tool for assessing orthorexia tendencies and behaviors, addressing the limitations of existing ON-specific measures. This study aimed to evaluate the psychometric properties of the Chinese version of the ONI.

**Methods:**

A total of 717 participants (Mage = 20.11 years, 78.66% female) completed the Orthorexia Nervosa Inventory (ONI) alongside the Chinese version of the Düsseldorf Orthorexia Scale (C-DOS). The ONI was translated into Chinese using the Brislin traditional translation model, following formal authorization from the original author. This translation process included literal translation, back translation, and cultural adaptation to ensure both linguistic and contextual fidelity. Item analysis was employed to assess item differentiation. Scale reliability was determined by measuring internal consistency. Furthermore, exploratory and confirmatory factor analyses were conducted to investigate and confirm the underlying factor structure and overall validity of the scale.

**Results:**

The Chinese version of the Orthorexia Nervosa Inventory (ONI) consists of 24 items across three dimensions. The overall Cronbach’s alpha coefficient for the scale was 0.956, indicating excellent internal consistency. The Cronbach’s alpha coefficients for the individual dimensions were 0.894, 0.933, and 0.848, respectively, demonstrating high reliability for each dimension. Additionally, McDonald’s *ω* was 0.957 for the entire scale, reflecting strong stability in internal consistency, with individual dimensions having McDonald’s *ω* coefficients of 0.895, 0.934, and 0.854. The Spearman-Brown split-half reliability coefficient was 0.931, and McDonald’s ω for the split-half reliability was also 0.931, indicating excellent consistency across the scale’s two halves. The test–retest reliability was 0.987, with a 95% confidence interval ranging from 0.978 to 0.993, suggesting excellent stability over time and strong consistency across different measurement points. All model fit indices fell within acceptable ranges, affirming the structural validity of the Chinese version. The results from both exploratory and confirmatory factor analyses further supported this conclusion.

**Conclusion:**

This study successfully translated and culturally adapted the ONI into Chinese, followed by a comprehensive evaluation of its psychometric properties. The findings demonstrate that the Chinese version of the ONI possesses strong reliability and validity. In the context of varying cultural backgrounds and dietary habits, this scale serves as a valid tool for assessing and screening the Chinese ON population.

## 1 Introduction

Orthorexia is originated from Greek *ortho* created by Bratman (1) and means correct appetite (2). Orthorexia Nervosa (ON) is an eating disorder which describes people engaging in extreme diet patterns for health, the main characteristics are the compulsive thinking and obsessive behavior for “healthy food.” During a 2022 consensus meeting attended by 47 eating disorders experts from 14 different countries, Orthorexia Nervosa (ON) was classified as a mental health disorder that is closely aligned with the DSM-5 category of ‘Feeding and Eating Disorders’ (F&ED) (3). The excessive focus on food quality and pursuit of “purity of food” may lead to the development of certain forms of eating disorders with harmful and counter-productive result. Studies find that ON may lead to some adverse outcomes like malnutrition and/or social dysfunction (1). Dieters with ON may experience nutritional deficiency result from ignoring the diversity of food groups, long term dietary restriction develop symptoms similar to severe anorexia such as osteopenia, anemia, hyponatremia, metabolic acidosis, pancytopenia, testosterone deficiency, and bradycardia (4, 5).

At present, ON has no widely accepted official definition and is not listed in official ICD-11 or DSM-V (2). Though, there’re amount of diagnostic criteria for ON have been proposed (3), most of them are criticized by researchers or have not been verified. As a result, there’s no standardized diagnostic criteria and treatment regime. The diagnostic criteria, classification, and underlying mechanisms of ON are still under discussion. At present, the possible treatment for ON is based on MTD model consisted of pharmacy, cognitive behavioral and nutritional intervention (5–7). In order to find reliable diagnostic criteria, researchers have developed many tools for ON measurement or diagnosis. For example, Orthorexia Self-Test (BOT) developed by Bratman and Knight (8) is widely used to diagnose ON, however, it’s criticized for the invalid psychometric characteristics. ORTO-15 was designed by Donini et al. (9) and translated into multiple language version (10–12) as a diagnostic tool for ON, while it was questioned for limitation like no clear validation of the tool, no standardization methods, and an excessive percentage of ON; Eating Habits Questionnaire (EHQ) developed by Gravers (13), though with high integrity, there’s controversy for its factor structure. Other scales are not widely applied yet, the quality and the validity need verification. On the other hand, content most of scales focus on knowledge, behaviors or emotions, however, physical impairments caused by ON are ignored, which are very important for clinic.

Orthorexia Nervosa Inventory (ONI) is developed by Oberle et al. (14) in 2020 which is a self-report questionnaire consisted of three dimensions: behaviors and preoccupation with healthy eating; physical and psychosocial impairments; and emotional distress, with a total of 24 items. The initial study reported the strong internal consistency level (total Cronbach *a* = 0.94, subscale Cronbach *a* > 0.86) and retest reliability (*r* > 0.86). What’s more, ONI is the first tool to assess physical impairment by ON, which had proved to be key factors of ON (14).

At present, the validity of the English version of ONI has been verified, but it has not been translated into Chinese version and applied to Chinese patients. Therefore, the purpose of this study is to translate the English version of ONI into Chinese and to examine the psychometric properties of the Chinese version of the ONI. By investigating its reliability, factor structure, and validity. Meanwhile In order to evaluate the criterion validity of the ONI, the Chinese version of the 10-item Düsseldorf Orthorexia Scale (C-DOS) was employed.

## 2 Methods

### 2.1 Original ONI

The ONI (14) is a self-report instrument comprising 24 items, designed to assess three latent dimensions: behaviors and fixation on healthy eating, physical and psychosocial impairments, and emotional distress. Respondents rate each item on a 4-point scale, where 1 indicates ‘not at all’ and 4 indicates ‘very ‘. Scale scores are derived by summing the items corresponding to each dimension, and an overall score is calculated by summing all item scores. The total score ranges from 24 to 96, with higher scores reflecting more pronounced orthorexia symptomatology.

### 2.2 Translation and culture adaption

After obtaining permission from the original authors of the Orthorexia Nervosa Inventory (ONI) to translate and contextually modify the questionnaire, we carefully adhered to prescribed guidelines throughout the process. A comprehensive forward- and back-translation process was employed to ensure accuracy and cultural relevance. The translation and cross-cultural adaptation were rigorously executed in strict accordance with the Brislin translation model, culminating in the creation of a Chinese version of the ONI (15, 16). The specific procedure involved several detailed steps.

#### 2.2.1 Forward translation

The original English version of the scale was independently translated into Chinese by two bilingual researchers on our team, both of whom are native Chinese speakers. These initial translations, labeled T1 and T2, were carefully reviewed and compared by the research team. Through in-depth discussions, any discrepancies were resolved, leading to the integration of these versions into a preliminary draft, designated as T3.

#### 2.2.2 Back translation

To ensure the accuracy of the translation, T3 was independently back-translated into English by two bilingual native English speakers who were unfamiliar with the original scale. These back-translations, referred to as E1 and E2, were synthesized into a single English version, ET3. This version was then sent to the original authors of the scale for feedback. Based on their input and further deliberations within the research team, the first finalized Chinese version, labeled C1, was produced.

#### 2.2.3 Cross-culture validation

In October 2023, the Chinese version C1 underwent a rigorous cultural adaptation process utilizing the Delphi method. A panel of seven experts in eating disorders—each holding an associate senior professional title, a master’s degree or higher, and over 15 years of professional experience—was convened. These experts evaluated the context, cultural relevance, and linguistic expression of the C1 items based on their clinical experience and theoretical knowledge, while also referencing the original scale. Following their review, the research team made the necessary adjustments to the questionnaire based on the experts’ feedback, resulting in the development of the revised Chinese version, C2.

### 2.3 Participants

Data for this study were gathered through an online questionnaire administered between March and May 2024. To guarantee the dependability of the analysis outcomes, the sample size for Exploratory Factor Analysis (EFA) should include a minimum of 120 cases, while Confirmatory Factor Analysis (CFA) requires at least 200 cases. This recommendation follows the guideline that the sample size should be 5–10 times greater than the number of variables (17). And considering a sample loss rate of 20% (18), the total sample size should be at least 384.

After receiving a detailed explanation of the study’s objectives and procedures, participants provided informed consent before voluntarily completing the survey. Importantly, no incentives were offered, ensuring genuine participation. The introductory page of the questionnaire provided a thorough overview of the study, explicitly informing respondents of their right to withdraw at any point by choosing not to submit their responses. Each survey session was designed to be brief, lasting approximately 10 min, with strict measures taken to guarantee complete anonymity; no personally identifiable information was collected. The study targeted adults who met specific criteria: participants were required to be of legal age (18 years or older), native speakers of Mandarin Chinese, and willing to provide informed consent. Those with visual or cognitive impairments that hindered their ability to complete the survey were excluded from the study.

### 2.4 Statistical software

For data entry and analysis, SPSS version 25.0, JAMOVI 2.3.28 and AMOS version 23.0 were utilized. Before the data analysis, we manually deleted the incomplete data.

### 2.5 Reliability analysis

Reliability serves as a key indicator of the accuracy and consistency of a measurement instrument in capturing the true characteristics of the variables being measured. It reflects the extent to which the measurement tool consistently produces stable and dependable results over repeated trials. In essence, high reliability indicates that the instrument reliably measures the intended variables with minimal error, ensuring that the observed results are a true representation of the measured object or variable. The greater the consistency in test outcomes, the lower the measurement error, and consequently, the higher the reliability of the instrument (19).

#### 2.5.1 Cronbach’s alpha

Cronbach’s alpha coefficient was computed to evaluate the internal consistency of the scale. A value of *α* > 0.70 was considered acceptable for demonstrating reliability, indicating that the items within each dimension were sufficiently correlated (20).

#### 2.5.2 Omega coefficient

In addition to Cronbach’s alpha, the omega coefficient was calculated to provide a more accurate assessment of internal consistency. The omega coefficient is particularly useful when the scale is multidimensional, as it accounts for inter-dimensional variability.

#### 2.5.3 Standard error of measurement (SEM)

The Standard Error of Measurement (SEM) was calculated to estimate the precision of the scale’s scores. SEM reflects the amount of error in measurement and was calculated using the formula: SEM = *σ*1 − *α*, where *σ* is the standard deviation of scores and *α* is the Cronbach’s alpha coefficient. Lower SEM values indicate greater precision and reliability of the scale.

#### 2.5.4 Split-half reliability

Split-half reliability was used to assess the internal consistency of the scale by dividing the items into two equal halves and comparing the scores from each half. The Spearman-Brown prophecy formula was applied to adjust for the fact that the split version uses only half the items, providing a reliability estimate for the full scale. A split-half reliability coefficient greater than 0.70 indicates good internal consistency.

#### 2.5.5 Test–retest reliability

Test–retest reliability of the scale was calculated using the intraclass correlation coefficient. To examine the stability of the scale over time, a test–retest reliability analysis was conducted. A subset of participants completed the scale twice, with a 2-week interval between administrations. The Pearson correlation coefficient was used to assess the strength of the relationship between the two sets of scores. A correlation greater than 0.70 was considered indicative of good stability.

### 2.6 Validity analysis

Several methods were employed to assess the validity of the adapted scale, ensuring that it accurately measures the intended construct and can discriminate between relevant groups.

#### 2.6.1 Independent samples t-test

To assess the discriminant validity, an Independent Samples t-Test was performed to compare scores between groups with different levels of the target construct. Significant differences (*p* < 0.05) would provide evidence for the scale’s ability to discriminate between relevant groups.

#### 2.6.2 Criterion validity

In order to evaluate the criterion validity of the ONI, the C-DOS was employed (21). The C-DOS demonstrated good internal consistency, with an ordinal alpha of 0.80, and solid test–retest reliability, with a coefficient of 0.77. The scale items are rated on a 4-point scale, ranging from 1 (“this does not apply to me”) to 4 (“this applies to me”), yielding total scores ranging from 10 to 40. Higher scores indicate more severe orthorexia symptoms.

#### 2.6.3 Exploratory factor analysis (EFA)

Exploratory Factor Analysis (EFA) was performed to explore the underlying structure of the scale. This analysis helped identify the number of factors and the items that loaded onto each factor. Principal Axis Factoring (PAF) was used for factor extraction.

#### 2.6.4 Confirmatory factor analysis (CFA)

This study employed Confirmatory Factor Analysis (CFA) to examine the factor structure of the scale and to validate the model proposed by the Exploratory Factor Analysis (EFA). CFA is a structural equation modeling (SEM) technique used to assess the fit between the data and a theoretical model. By using CFA, the construct validity of the scale was tested, the relationships between latent variables and observed variables were clarified, and the overall model fit indices *χ*^2^/df, RMR, CFI, RMSEA, GFI, AGFI were evaluated.

### 2.7 Item analysis

Item analysis was conducted to evaluate the performance of individual items within the scale and to identify problematic items that may need modification (27).

#### 2.7.1 Item-total correlation

Item-total correlation was calculated to assess the relationship between each item and the total scale score, excluding the item itself. Items with a correlation greater than 0.30 were considered to contribute well to the overall scale, while items with lower correlations were flagged for potential revision.

#### 2.7.2 Item response theory (IRT)

Graded Response Model (GRM) is employed to conduct Item Characteristic Curve (ICC) analyses for each item of the scale, aiming to evaluate the applicability and discrimination of each rating category across varying ability levels.

## 3 Results

### 3.1 Demographics

In this study, 720 questionnaires were distributed, and 717 were successfully retrieved, yielding an effective response rate of 99.72%. 153 of them (21.34%) were male and 564 (78.66%) were female. Average scale score is 72.063 ± 14.677. The demographic information collected includes age, sex, marital status, education level, residence and BMI. Additional social demographic details are presented in Table 1.

**Table 1:** Independent samples t-test results for gender.

		Statistic	df	*p*
Total score	Student’s t	2.51^a^	712	0.012
	Welch’s t	2.32	217	0.021

H_a_ μ _male_ ≠ μ _female_.

a Levene’s test is significant (*p* < 0.05), suggesting a violation of the assumption of equal variances.

**Table 2:** Independent samples t-test results for medical education background.

		Statistic	Df	*p*
Total score	Student’s t	3.13^a^	712	0.002
	Welch’s t	2.67	87.2	0.009

H_a_ μ _1_ ≠ μ _2_.

a Levene’s test is significant (*p* < 0.05), suggesting a violation of the assumption of equal variances.

^*^μ_1_ represents individuals without a medical background, while μ_2_ represents individuals with a medical background.

### 3.2 Scale translation and cross-cultural adaptation

In accordance with the linguistic and cultural nuances of China, the initial draft of the Chinese version was carefully reviewed and adjusted, considering semantics, idiomatic expressions, and cultural concepts. First, the original scale was translated from English to Chinese using forward translation. Subsequently, the instruments were translated into English. Finally, the authors scrutinized the phraseology of both the English and Chinese versions and compared them with the original English version, with the objective of identifying uncertainties and correct inconsistencies. Both researchers and linguists approved the Chinese version of the study prior to the research.

### 3.3 Reliability

#### 3.3.1 Cronbach’s alpha and omega coefficient

Cronbach’*α* coefficients for each dimension and total scale were calculated, all dimensions showed good reliability with Cronbach’s *α* = 0.894 and McDonald’s *ω* = 0.895 (Dimension1); Cronbach’s *α* = 0.933 and McDonald’s *ω* = 0.934 (Dimension 2); Cronbach’s *α* = 0.848 and McDonald’s *ω* = 0.854 (Dimension 3), and the total scale also shows excellent reliability with Cronbach’s *α* = 0.956 and McDonald’s *ω* = 0.957.

#### 3.3.2 SEM

According to the formula SEM = SD × √(1 − *α*), we obtain SEM of 3.08. The scale consists of 24 items presented in a 4-point Likert-type format. For a scale with a total score of 96, an SEM of 3.08 represents a measurement error of approximately 3.21%. This is considered a relatively ideal value, indicating that the measurement error is acceptable.

#### 3.3.3 Split-half reliability

Value of Spearman-Brown split-half reliability coefficient is 0.931, which indicates the high consistency across different parts of scale and shows the good reliability.

#### 3.3.4 Test–retest reliability

The test–retest reliability for DOS scores was found to be 0.987. 95% CI from 0.978 to 0.993.

### 3.4 Validity

#### 3.4.1 Independent samples t-test

In order to learn about the influence of socio-demographic variables, we made the independent samples t-test and found that the gender and medical education background had significant impact on result (*p* < 0.05) while BMI did not (Tables 1–3).

**Table 3:** Correlation matrix for ONI total scores and BMI.

		Total score	BMI
Total score	Pearson’s *r*	—	
	Df	—	
	*p*-value	—	
BMI	Pearson’s *r*	0.028	—
	Df	712	—
	*p*-value	0.461	—

#### 3.4.2 Criterion validity

C-DOS was used to analyze criterion validity of C-ONI and the total score of C-ONI and C-DOS was positively correlated (*r* = 0.87, *p* < 0.001).

#### 3.4.3 EFA

**Figure 1:**
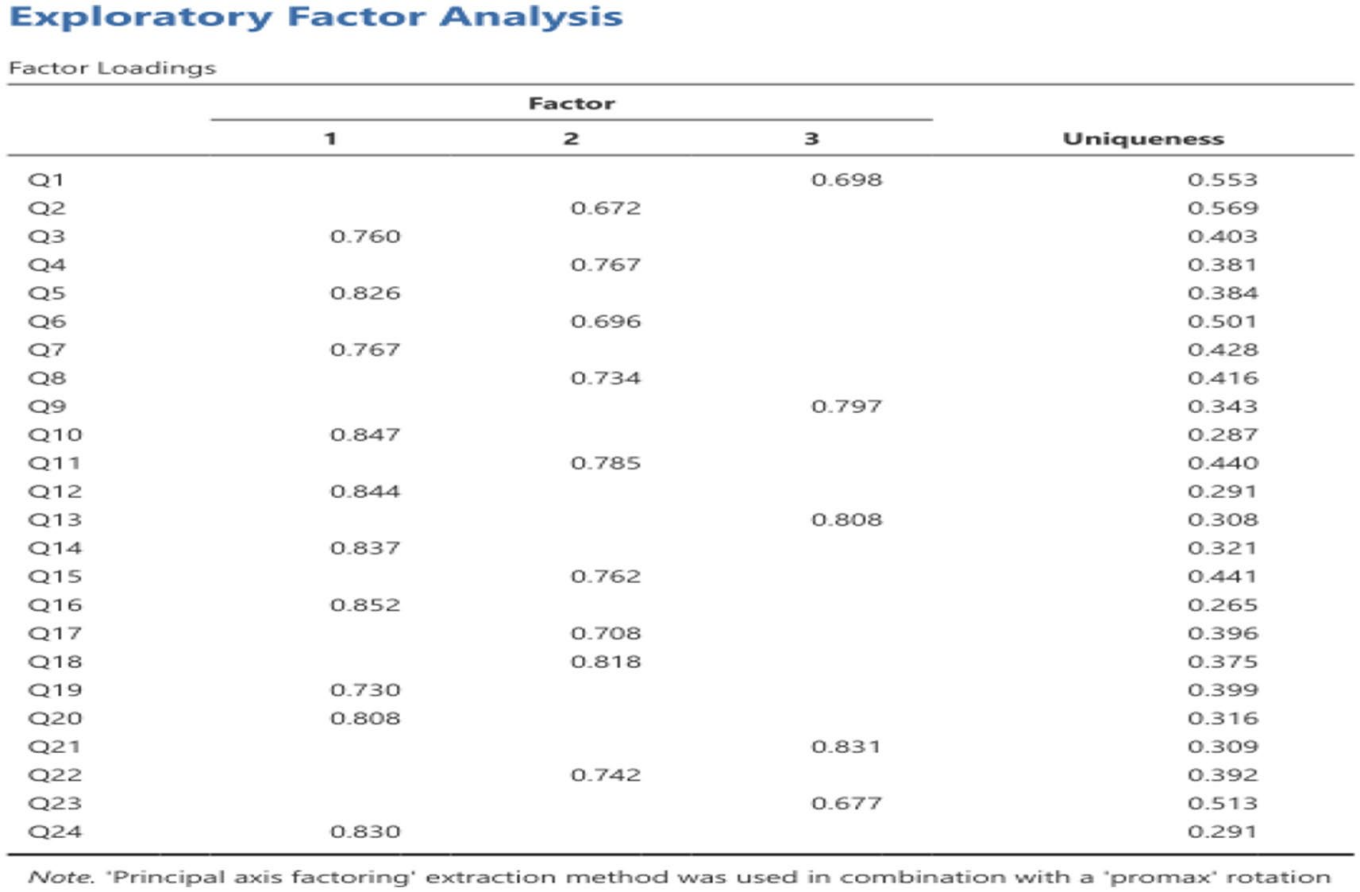
Factor loadings of each item in the Chinese version ONI.

The KMO value is 0.949, >0.6, satisfied with the requirement of factor analysis suggesting that the factor analysis can be used for data. Bartlett’s test of sphericity also showed the data is suit for factor analysis (*p* < 0.05). The Principal Axis Factoring method was adopted in combination with Promax rotation. The results indicated that the 24 measurement items were distributed across three latent factors, with significant factor loadings explaining the primary variance structure of each variable. The key findings are summarized as follows: Factor 1 is primarily defined by significant loadings from items Q3, Q4, Q5, Q7, Q10, Q12, Q13, Q14, Q16, Q19, and Q24 (factor loadings ranging from 0.730 to 0.852); Factor 2 is characterized by significant loadings from items Q2, Q6, Q8, Q11, Q15, Q17, Q18, and Q22 (factor loadings ranging from 0.672 to 0.818); Factor 3 is mainly defined by significant loadings from items Q1, Q9, Q20, Q21, and Q23 (factor loadings ranging from 0.677 to 0.831). The factor loading for each item is shown in Figure 1.

#### 3.4.4 Confirmatory factor analysis

**Figure 2:**
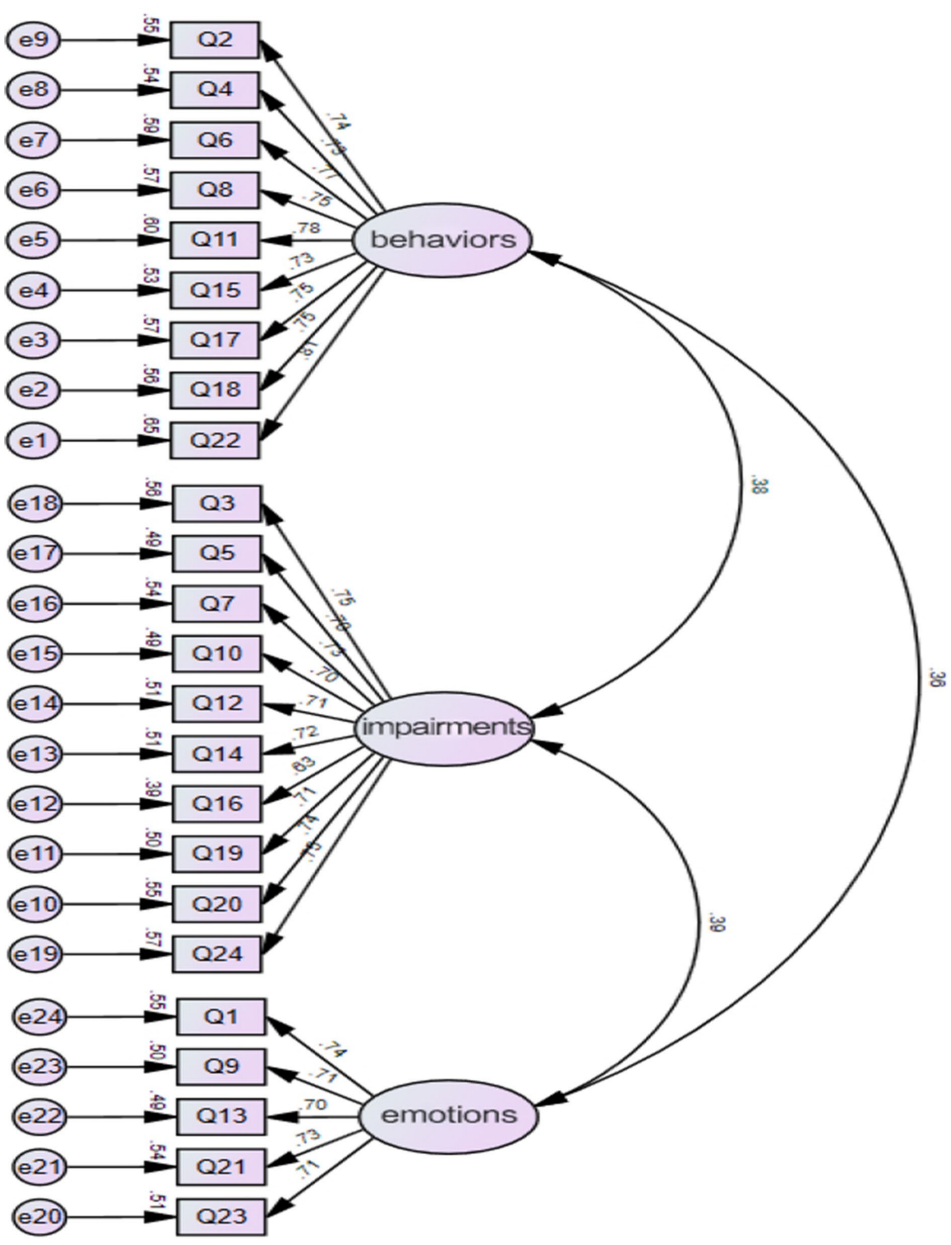
Standardized three-factor structural model of the Chinese version of the ONI (*N* = 428).

The model was constructed using Amos 23.0 software, and confirmatory factor analysis (CFA) was conducted on the survey data to derive the structural equation model, as illustrated in Figure 2.

The goodness-of-fit indices for the models are presented in Table 3. In conclusion, the Chinese version of the ONI demonstrates good structural validity.

### 3.5 Item analysis

The purpose of item analysis is to determine whether the items in the questionnaire or scale are valid and appropriate.

#### 3.5.1 Item-total correlation

All items demonstrating correlations exceeding 0.5 and the majority approaching or surpassing 0.7, which indicates that most items significantly contribute to the overall construct of the scale, underscoring strong construct validity.

#### 3.5.2 ICC analyses

All 24 ICC curves illustrate the probability of selecting different rating categories across the spectrum from low to high ability segments. Below is a detailed analysis of these curves.

##### 3.5.2.1 Relationship between ability and selection probability

All 24 curves demonstrated that as participants’ abilities increased, the probability of selecting higher rating categories (e.g., Category 3 and Category 4) progressively rose, while the probability of selecting lower rating categories (e.g., Category 1 and Category 2) decreased. This trend indicates that the scale exhibits strong ability discrimination, effectively distinguishing participants across different ability levels.

##### 3.5.2.2 Smoothness and monotonicity of the curves

The majority of the curves displayed smooth and monotonically increasing transitions, aligning with GRM expectations. This indicates that the transitions between rating categories are logical and free from abrupt changes or plateau regions, thereby reinforcing the scale’s measurement precision and reliability.

##### 3.5.2.3 Thresholds and category spacing

The intersections of the curves were appropriately distributed, suggesting that the thresholds between rating categories are well-calibrated and can clearly differentiate each category. Notably, in the low and high ability intervals, the differences in the probability of selecting each rating category were substantial, reflecting the scale’s excellent discrimination in these extreme ability segments.

##### 3.5.2.4 Discrimination in the moderate ability range

However, within the moderate ability range (approximately around an ability value of 0), some curves exhibited relatively gradual transitions, particularly between Category 2 and Category 3, showing minimal differences in selection probabilities. This may hinder participants in the moderate ability range from distinctly differentiating these rating categories, potentially compromising measurement precision. This observation suggests that, while most curves meet expected standards, the discrimination within the moderate ability range may be insufficient and warrants further optimization.

##### 3.5.2.5 Applicability of rating categories

Overall, the performance of all 24 curves indicates that the spacing between rating categories is appropriate, effectively reflecting varying levels of participants’ abilities. Through GRM model fitting, we confirm that the scale’s design aligns with expectations and provides high measurement precision across most ability segments.

##### 3.5.2.6 Model fit

Following GRM analyses for all items, the scale demonstrated robust model fit. The transitions between categories were smooth, and there were no significant overlaps or issues in distinguishing rating categories.

In summary, the ICC curves of the scale exhibit optimal discrimination among rating categories, particularly within the extreme ability segments (low and high abilities), where the rating categories effectively differentiate participants with varying ability levels. Nonetheless, in the moderate ability range, some curves display gradual transitions and insufficient discrimination between categories, which may impact measurement precision in this segment.

## 4 Discussion

The present study aimed to investigate the psychometric properties of the ONI within a sample of Chinese respondents, providing valuable insights into its factorial structure, internal consistency, as well as its convergent and criterion validity.

Eating disorders manifest differently in different cultures and social preferences, and research has been conducted to support the idea that eating disorders manifest differently in Asian countries. More research is needed to investigate the cross-cultural validity of ON and how it manifests differently in different culture background (1, 22, 23). The development of C-ONI can help researchers investigate the different prevalence rates of ON across cultures and enhance the identification and screening of the disease.

### 4.1 The Chinese version of the scale has suitable reliability

This study assessed the reliability of the Chinese version of the ONI from different aspects, and most of them show the high reliability of the scale. Cronbach’s *α* and Omega Coefficient of all dimensions and the whole scale suggest that the items across all dimensions are well-aligned and contribute effectively to the construction. Split-half reliability also showed the high consistence among different parts of the scale. Test–retest reliability indicates the stability of the scale over time and its consistency with the measured object, suggesting that the measurement results are highly reliable and suitable for long-term or repeated use.

Overall, the scale is highly reliable, with stable internal consistency across all dimensions and minimal impact from item deletions, confirming its suitability for measuring the intended construction.

### 4.2 The Chinese version of the scale has suitable validity

In this study, the validity of the scale was analyzed and assessed using both content validity and construct validity. Content validity measures how well the scale items align with the intended measurement objectives and requirements, while construct validity assesses the extent to which the scale’s theoretical framework is reflected in the observed measurement outcomes (24). The I-CVI of the Chinese version ONI was between 0.857 and 1.000, and the S-CVI was 0.917, which was higher than the reference value of content validity and good content validity (25). Suitable for assessing the Chinese population.

To assess the construct validity of the Chinese scale, both Exploratory Factor Analysis (EFA) and Confirmatory Factor Analysis (CFA) were employed. It is widely accepted that ideal structural validity should meet the following criteria: The exploratory factor analysis followed the criteria that (1) the extracted factors should collectively explain at least 40.00% of the total variance, and (2) each item should demonstrate a high factor loading (>0.400) on one factor while showing low loadings on other factors. The results indicated that Principal Axis Factoring (PAF) with Promax rotation was applied, and only items with individual factor loadings greater than 0.40 were retained. Ultimately, three common factors were extracted, which collectively accounted for a substantial portion of the variance. The factor loadings across the component matrix were all above 0.5 on their respective dimensions, demonstrating strong associations between items and their corresponding factors. Three common factors were ultimately extracted, collectively accounting for 61.100% of the total variance (26). The results of CFA showed that *χ*^2^/df = 1.738, RMSEA = 0.924, RMR = 0.027, CFI = 0.965, GFI = 0.924, AGFI = 0.908. Convergent validity refers to the size of the factor loading coefficient of the corresponding variable reflected by each item, which is mostly calculated by AVE and CR. The standardization of each variable in this study is greater than 0.7, the AVE value is greater than 0.5, and the CR value is greater than 0.8. Therefore, the convergent validity test of the research data in this article is qualified. On the other hand, Item-Total Correlation shows the existing item structure is well-supported and should be retained. Only items with relatively lower correlations may require minor refinement to enhance their effectiveness. Overall, the scale exhibits excellent construct validity, ensuring that the total score effectively encapsulates the intended measurement objectives.

## 5 Limitations

This study has several limitations. Firstly, we recruited a non-clinical sample, which may affect the generalizability of the findings. Future surveys will be conducted on a broader group of people. Secondly the scale used in this study does not have a predefined diagnostic threshold, and therefore, ROC analysis cannot be performed. The scale is designed as a continuous measurement tool and is not intended for binary classification based on a fixed cutoff (i.e., distinguishing between positive and negative cases). Since ROC analysis requires calculating sensitivity and specificity at various thresholds, traditional ROC analysis is not applicable in the absence of a clear diagnostic threshold. The third point is, through calculation, we found that the floor effect of most items exceeded 20%, which is primarily attributed to differences in scale design and participants’ ability levels, rather than a lack of item difficulty or discrimination. Therefore, we can confirm that the scale items are effective in terms of design and measurement properties. To reduce the floor effect, further adjustments to the scoring criteria or an increase in the number of items will be necessary in the future. In addition, the primary purpose of this study was the cultural adaptation of the scale. Therefore, during the collection of demographic data, a more diverse range of demographic information was not considered. As a result, only limited hypotheses regarding demographic variables were tested. In future applications of the scale, more in-depth research can be conducted.

## 6 Conclusion

This study concentrated on translating and culturally adapting the Orthorexia Nervosa Inventory (ONI) into Chinese, followed by a thorough evaluation of its psychometric properties. The findings demonstrate that the Chinese version of the ONI exhibits strong reliability and validity. In the context of diverse cultural backgrounds and dietary habits, this scale provides a reliable tool for assessing and screening the Chinese ON population. Additionally, it lays the groundwork for future intervention studies targeting this group.

## Data Availability

The data supporting the findings of this study are available from the corresponding author upon request.
